# Prospective of Genomics in Revealing Transmission, Reassortment and Evolution of Wildlife-Borne Avian Influenza A (H5N1) Viruses

**DOI:** 10.2174/138920211797904052

**Published:** 2011-11

**Authors:** Fumin Lei, Weifeng Shi

**Affiliations:** 1 Key Laboratory of the Zoological Systematics and Evolution, Institute of Zoology, Chinese Academy of Sciences, Beijing 100101, China; 2 The Conway Institute of Biomolecular and Biomedical Research, University College Dublin, Belfield, Dublin 4, Ireland

**Keywords:** Avian Influenza H5N1, reassortment, transmission, genomic, phylogeny, wild bird.

## Abstract

The outbreak of highly pathogenic avian influenza (HPAI) H5N1 disease has led to significant loss of poultry and wild life and case fatality rates in humans of 60%. Wild birds are natural hosts for all avian influenza virus subtypes and over120 bird species have been reported with evidence of H5N1 infection. Influenza A viruses possess a segmented RNA genome and are characterized by frequently occurring genetic reassortment events, which play a very important role in virus evolution and the spread of novel gene constellations in immunologically naïve human and animal populations. Phylogenetic analysis of whole genome or sub-genomic sequences is a standard means for delineating genetic variation, novel reassortment events, and surveillance to trace the global transmission pathways. In this paper, special emphasis is given to the transmission and circulation of H5N1 among wild life populations, and to the reassortment events that are associated with inter-host transmission of the H5N1 viruses when they infect different hosts, such as birds, pigs and humans. In addition, we review the inter-subtype reassortment of the viral segments encoding inner proteins between the H5N1 viruses and viruses of other subtypes, such as H9N2 and H6N1. Finally, we highlight the usefulness of genomic sequences in molecular epidemiological analysis of HPAI H5N1 and the technical limitations in existing analytical methods that hinder them from playing a greater role in virological research.

## INTRODUCTION

A highly pathogenic avian influenza (HPAI) H5N1 virus was firstly isolated from Guangdong, China in 1996 [1]. In the intervening years, HPAI has resulted in disease outbreaks in over 63 countries across Asia, Europe and Africa and ongoing laboratory surveillance has detected its circulation in 9 countries in 2011 (http://www.oie.int/en/animal-health-in-the-world/update-on-avian-influenza/2010/). Wild birds are considered as natural hosts for all subtypes of avian influenza (AI) viruses [2-4]. HPAI H5N1 virus has been identified from over 120 wild bird species in nearly 30 countries (http://www.birdlife.org/). More importantly, it has caused a total of 553 confirmed cases of human infection, 323 people of whom died (http://www.who.int/csr/ disease/avian_influenza/country/cases_table_2011_04_21/en/index.html). It has also been isolated from other mammals, such as pigs, tigers, leopards, civets, cats, dogs, stone martens, pikas and donkeys. Therefore, it poses a serious threat to poultry industry, wild bird conservation and global public health.

The genome of H5N1 virus is composed of eight segmented RNA fragments of negative sense [5]. The first three segments encode viral polymerase basic proteins PB2, PB1 and PA respectively. The fourth viral segment encodes hemagglutinin (HA), which is an important surface glycoprotein and the major antigen of the virus. HA is responsible for attaching the virions to the host sialic acid receptors on respiratory epithelia and is a critical determinant of pathogenicity. Nucleoprotein (NP), encoded by the fifth segment, binds to and encapsidates viral RNA in the infected cell nucleus. The sixth segment encodes another important surface-exposed glycoprotein, neuraminidase (NA). The major role of this protein is to release newly produced viral particles by cleaving sialic acid residues on host cells facilitating further infection. Mutations in the NA segment (eg: H275Y) are also associated with decreased antiviral susceptibility to the NA inhibitor drug class (oseltamivir, zanamivir). The seventh viral segment encodes two different proteins M1 and M2 by using alternative reading frames and the adamantane class of drugs target this proton channel in the virus surface. The final viral segment encodes two nonstructural proteins NS1 and NS2 due to alternatively splicing events.

If a single host is infected by two different subtypes of AI virus, it is possible that newly assembled viral particles will be created from segments whose origin is mixed, some coming from one subtype and some coming from another. This genetic reassortment plays a vital role in the origin of newly emerging pathogens of humans and animals as evidenced by the recent emergence of the swine-origin influenza A (H1N1) 2009 virus [6]. Phylogenetic analysis of viral sequences is a standard way to study the genetic reassortment by comparing different phylogenetic trees constructed using all eight genomic segments. Previous studies have identified many genetic reassortment events in the HPAI H5N1 viruses [7-9].

With recent advances in sequencing technology and the associated decrease in cost, more and more virologists use sequencing for epidemiological purposes [10-12]. AI sequences data resources, such as the NCBI Influenza Virus Resource (http://www.ncbi.nlm.nih.gov/genomes/FLU/FLU. html) [13] and the Global Initiative on Sharing All Influenza Data (GISAID; http://platform.gisaid.org/) database [14], have provided the data platform for the storage and distribution of viral genomic information within the scientific community [12]. Phylogenetic analysis has played a more and more important role in epidemiological studies of AI viruses. Analyses based on partial genomic segments, predominantly based on the major epitopes HA and NA, or the whole genome sequences have provoked a reassessment of the origins, global circulation patterns, reassortment and evolution of the AI viruses [7-9,15]. This has led to profound insight into the global patterns of circulation of the viruses and improved global health protection strategies based on the interruption of transmission [12].

In this paper, we have reviewed the role of viral genome sequences in revealing the mechanisms of the widespread and circulation of H5N1 viruses among wild life populations. We also reviewed the inter-host transmission and inter-subtype reassortment of H5N1 viruses. Finally, we have highlighted the technical limitations in existing analytical methods that hinder genomic sequences from playing a greater role in virological research and the usefulness of genomic sequences in molecular epidemiological analysis of HPAI H5N1.

## 1 GENOMIC SEQUENCES IN TRACING THE SOURCE AND UNDERSTANDING THE ROLE OF WILD BIRDS IN THE H5N1 SPREAD

The United Nations Food and Argiculture Organisation (FAO) has established recommendations for the nomenclature for clade determination and genotyping by phylogenetic analysis based on the viral HA gene, e.g. Clade 2.2, clade 2.3, etc [16]. Vijaykrishna and colleagues (2008) addressed the evolutionary dynamics of HPAI H5N1 viruses through the estimation of divergence times of gene segments of major reassortants and population dynamics analyses of the viruses in poultry [17]. Thirteen different nodes were identified, eg. I: Gs/GD, II: X-series, III: Clades 1, 2, 8, 9; IV: Clade 1; V: Vietnam, Thailand, Malaysia (VTM)+ precursor; VI: VTM; VII: Indonesia+precursor; VIII: Clade 2.1 (Indonesia); IX: Clade 2.2 (Qinghai lineage); X: Clade 2.3; XI: Clades 2.3.1, 2.3.2; XII: Clades 2.3.3, 2.3.4; XIII: Clade 2.3.4 (Fujian-like).

The prototype virus of HPAI H5N1 virus was isolated from Guangdong, China in 1996 [1] and was responsible for the influenza outbreak in Hong Kong Special Autonomous Region (SAR) in 1997 [15]. HPAI H5N1 gradually became established among poultry in different regions of China and developed into several phylogenetically different lineages or genotypes [7-9]. In late 2003 and early 2004, infections caused by HPAI H5N1 viruses were reported in several neighboring Asian countries (http://www.who.int/csr/ disease/avian_influenza/ai_timeline/en/index.html). In May 2005, H5N1 virus caused an outbreak in migratory waterfowl in Qinghai Lake, western China [18, 19]. Subsequently, H5N1 influenza outbreaks were reported in Xinjiang Uygur and Tibet Autonomous regions of China, Kazakhstan, Mongolia, Siberia of Russia, Turkey, Romania, Croatia, the United Kingdom and Ukraine in 2005. In 2006, more European countries reported HPAI H5N1 outbreaks in poultries and wild birds and in February 2006, the first outbreak of HPAI H5N1 virus in Africa was reported in Nigeria (http://www.who.int/csr/disease/avian_influenza/ ai_timeline/en/index.html). Reid and co-workers (2010) reported the first incursion of HPAI H5N1 viruses of clade 2.3.2 into European poultry that infected Romanian domestic poultry and Bulgarian wild birds, which represented the most westerly spread of clade 2.3.2 viruses identified to date [20]. So far, HPAI H5N1 virus has caused outbreaks in more than 63 countries of Eurasia and Africa since 2003 and circulation is ongoing in 2011 (http://www.who.int/csr/disease/avian_ influenza/ai_timeline/en/index.html).

Several mechanisms have been proposed to explain the rapid spread of HPAI H5N1 viruses in Eurasia and Africa, two of which are the poultry transmission model [21] and the bird migration model [22, 23]. Gautheir-Clerc *et al*. (2007) suggested that human movement of domestic poultry was the main agent of global dispersal of the virus into Africa and Europe [21]. Phylogenetic studies also supported that clade 2.3.2 viruses established in Vietnam and southern China in 2004 and advanced northwards along poultry trading routes [24]. In addition, the introduction of the HPAI H5N1 viruses into Nigeria was also found to be associated with the legal and/or illegal poultry trade [25].

The bird migration model is favored by most reports as wild birds are considered natural hosts for low pathogenic AI viruses and they harbor all subtypes of AI viruses [2-4]. Apart from phylogenetic analysis, this mode of transmission has also been supported by findings obtained using other independent techniques. For example, results from satellite tracking and remote sensing related the outbreaks of HPAI H5N1 to migratory birds [26, 27]. A space-time cluster analysis also showed that the spread of HPAI H5N1 viruses and bird migratory routes correlated well [28]. Nonetheless, this view is still controversial [29-31] and a few studies have given opposing results which do not support this mechanism of HPAI transmission [32].

Phylogenetic analysis of the viral genome sequences has provided a lot of evidence that wild birds are implicated in the spread of HPAI H5N1 viruses from southern China to Africa along the migratory flyways. For example, the sequences of the Qinghai Lake isolates from 2005 have been traced to one migratory duck isolate from Poyang Lake, Jiangxi Province [33]. The movement of clade 2.2.2 between Lhasa in Tibet and Qinghai was consistent with the migration route of bar-headed geese along the Central Asian flyway [27,34]. The introduction of the HPAI H5N1 virus from Qinghai Lake to western Siberia was suggested to be caused by migratory birds revealed by the viral genomic sequencing analysis [35]. The virus spread from Russia to the Black Sea demonstrated a significant correlation with the migratory pathway of ducks [36]. In addition, phylogenetic analysis showed that the HPAI H5N1 outbreak in Sweden in 2006 was also associated with the long distance migration of the wild birds [37]. That full genome comparison of the African AI cases and other earlier worldwide isolates demonstrated a high genetic similarity also indicated the circulation within Africa through migratory birds’ migration [38]. 

Based on the bird migration model, water birds such as the great-crested grebe (*Podiceps cristatus*), tufted duck (*Aythya fuligula*), whooper swan (*Cygnus cygnus*) and black-headed gull (*Chroicocephalus ridibundus) *appear to be key to the widespread dissemination of subclade 2.3.2 viruses, and the bar-headed goose and ruddy shelduck, two migratory hosts for HPAI H5N1 along the Central Asia Flyway, emerged as potential vectors for the movement of clade 2.2.1 and clade 2.2.2 viruses (Newman *et al*. unpubl.). The bar-headed goose isolates from this outbreak were found sharing PB2 genes common to HPAI H5N1 circulating in live bird markets in Tibet [39].

## 2 A CASE STUDY: HPAI H5N1 VIRUSES FROM THE QINGHAI LAKE

From the first H5N1 outbreak among waterfowl populations in 2005 at Qinghai Lake, four isolates were sequenced. Phylogenetic analysis showed that five of the eight genomic segments (M, PA, PB1, PB2, and NS) were closely related to a Hong Kong isolate (A/peregrine falcon/HK/D0028/04). This suggested that the viruses might be created from reassortants that originated in birds over-wintering in Southeast Asia [19]. Another independent study showed that the HA and NA genes of the Qinghai isolates and other H5N1 viruses from poultry in Fujian, Guangdong, Hunan and Yunnan provinces from 2005 were similar to the H5N1 virus A/Chicken/Shantou/4231/2003, while the internal genes were closely related to H5N1 viruses from poultry described in southern China during 2005 (e.g. A/Chicken/Shantou/810/2005). This indicates that the viruses might be transmitted to Qinghai Lake from poultry in southern China *via *a single introduction [18].

After the 2005 out break, Kilpatrick* et al*. (2006) integrated phylogenetic relationships of the identified viral sequences, migratory bird movements, trade in poultry and wild birds to determine the pathway of the introduction events, and found that an important factor was the synergistic spread of H5N1 by poultry and wild birds [40]. Based on this, Kilpatrick *et al*. (2006) suggested that the most effective strategy to prevent H5N1 from cross hemisphere circulation would be even stricter controls or outright bans on illegal trade of poultry and wild birds [40].

In 2006, three viruses (BHGs/QH/F/06; GBHGull/ QH/3/06; Swan/QH/01/06) were isolated from three different wild bird species (bar-headed goose, great brown-headed gull and whooper swan) respectively from Qinghai Lake. Results from both phylogenetic analysis of these viruses and the hosts’ ecology suggested that these H5N1 viruses may be transported across different host species and spread into or out of Qinghai Lake through their migration [41].

Why did H5N1 viruses re-emerge in 2006 in Qinghai? Were these viruses transmitted from outside or did they re-emerge from a local niche? To answer these questions, Wang *et al*. (2008) conducted further phylogenetic analyses of the H5N1 viruses isolated from this region in both 2005 and 2006. Their results suggested that the Qinghai AI viruses from 2006 (QH06) most probably came from the flyway of migratory birds other than directly from the Qinghai cases from 2005 (QH05) [42]. They speculated that the AI QH05 strain may travel across the flyways spreading into Russia, and then transfer to the Mediterranean and European regions, then back to Qinghai Lake through wild bird migration. Genetic drift over the intervening year gave rise to the QH06 reintroduction in 2006.

Importantly, H5N1 viruses were also detected and genetically characterized from Qinghai Province in 2007 and demonstrated that two sequences were found more closely related to H5N1 viruses from Egypt, Togo, Ghana, and Nigeria in 2007. Therefore, migratory birds serving as vectors were the most parsimonious explanation for disseminating H5N1 strains *via *their overlapping flyways [39].

No viruses were isolated in 2008 in Qinghai Lake, but in both 2009 and 2010, H5N1 viruses were again described in the Qinghai aquatic wildfowl. Phylogenetic analysis of the HA sequences revealed that they were most closely related to clade 2.3.2 viruses from wild birds in Hong Kong and Japan during 2007-2008 [43]. This suggested that the 2009 and 2010 Qinghai strains were different from the QH05 virus of clade 2.2 [43]. They were also found closely related to those identified from Mongolia and Uvs Nuur Lake in 2009 [43-45]. Once again, this indicated that most viruses in the Qinghai Lake region might be transmitted by wild birds along the migration flyway [45].

The HPAI viruses described during 2009 and 2010 belonged to clade 2.3.2 and the HA cleavage site in these viruses was PQRERRRKRG, however in clade 2.2, an addition of a lysine residue (and consequent increase in charge) was described: PQRERRRKKRG [43,45]. The NA genes of the 2009 and 2010 isolates had a deletion of 20 amino acids at residues 49-68 in the stalk region. None of the detected amino acid substitutions in NA proteins were previously known to be associated with conferring decreased susceptibility to the adamantane or NAI class of anti-virals. Unlike past Qinghai Lake strains of clade 2.2, the Qinghai H5N1 strains from 2009 did not have an E627K substitution in the PB1 protein and furthermore the NS1 had a deletion of 5 amino acids at residues 80-84, which was commonly observed in HPAI H5N1 viruses that were circulating in Southeast Asia [43].

This review of the H5N1 viruses in wild birds described to date at Qinghai Lake suggests that they were mostly circulated by wild birds through the Central Asian flyway, and that HPAI pathotypes still exist at the lake and are still undergoing evolution. 

## 3 INTER-HOST TRANSMISSION OF HPAI H5N1

### 3.1 Transmission of HPAI H5N1 to Humans

In May 1997, an H5N1 virus was isolated from a 3-year-old boy from Hong Kong SAR for the first time [46-48]. Since then, H5N1 has been reported infecting humans in 15 countries, such as China [49-53], Vietnam [54, 55], Thailand [56, 57], Indonesia [58, 59], Cambodia, Turkey [60], Bangladesh [61] and Egypt [62, 63]. As of May 13, 2011, a total of 553 confirmed cases and 323 fatal cases of human infection with H5N1 have been reported worldwide (http://www.who.int/csr/disease/avian_influenza/country/cases_table_2011_04_21/en/index.html). 

Phylogenetic analysis of the genomic sequences of the human cases has been widely used to trace the origin and evolution of these HPAI pathotypes. The majority of the genomic sequences of the human H5N1 strains were reported to be derived from avian strains [47, 51, 56-58, 64-67]. For example, the genomic sequences responsible for the first human infection with H5N1 were found to be all avian-like [47, 64]. Although the human H5N1 isolates from Hong Kong SAR from 2002 were still of avian origin and they were closely related to the genotype Z and Z^+^ viruses, their internal proteins had a different origin with the H5N1 viruses that caused the first known case of human infection in Hong Kong in 1997 [65]. Phylogenetic analyses have also shown that all the eight segments of the human H5N1 strains from Thailand, Indonesia and other Asian countries from 2004 and 2005 were closely related to the avian isolates of genotype Z [56, 58, 66]. Therefore, human infection with H5N1 virus is most likely to be associated with direct or indirect contact with infected birds or wildfowl [60, 68, 69], although the possibilities of inter-personal transmission of HPAI H5N1 and environment-to-human transmission still exist [57, 58, 67, 69, 70].

Genetic analysis of specific amino acid mutations in the viral genome has also provided insight into the evolution and variation associated with the host-shift, drug-resistance and virulence of the viruses. For instance, the first HPAI H5N1 isolate from human from Hong Kong in 1997 possessed the RERRRKK motif at the basic cleavage site of the HA protein, which is considered a sign of HPAI viruses [71]. The first drug-resistant H5N1 virus whose NA protein had a histidine-to-tyrosine substitution at position 275 (N1 numbering) was isolated from a Vietnamese girl in 2005 [72] and this mutation has been reported to confer resistance to oseltamivir [73,74]. In particular, it was reported that this mutation emerged during the anti-viral treatment [75]. Viruses with the S294N mutation in the NA protein were isolated from humans from Egypt in late 2006 [76] and this amino acid substitution has been reported to reduce the susceptibility of the viruses to oseltamivir [72]. Similarly, viruses with the S31N mutation in the M2 protein, which is associated with amantadine resistance [77], have been isolated from humans from Hong Kong [65]. The D627K amino acid substitution in the PB2 protein was reported to increase the virulence of H5N1 viruses in mice [78, 79] and it has been observed in some human strains from Thailand [56] and Egypt [63]. More recently, it was also reported that a single mutation at position 192 or a double mutation at positions 129 and 151 of the HA protein could have increased the human-type receptor specificity of HPAI viruses that newly emerged in birds in Egypt [80].

### 3.2 Transmission of HPAI H5N1 to Pigs

Pigs have receptors that correspond to the AI-specific α-2,3-NeuAcGal sialic acid linkage and human influenza-specific α-2,6-NeuAcGal sialic acid linkage [81,82]. Therefore, they are regarded as a potential “mixing vessel” for avian and human influenza and the main intermediate host for AI viruses to make the appropriate genetic changes in order to infect humans [83-85]. However, there was no evidence that pigs had transmitted wholly AI viruses of H5N1 and other subtypes to humans [86]. It is reported that the susceptibility of domestic pigs to HPAI H5N1 is low [87] and the HPAI H5N1 viruses are not transmitted among pigs under experimental conditions [88]. A field study showed that no sera positive for H5 was detected in samples collected from Fujian Province, China in 2004 and 2007 [89]. In addition, the swine H5N1 isolates were less virulent to mice than avian isolates [90]. 

However, HPAI H5N1 has been isolated from pigs from China [91-93] and Indonesia [94]. Genetic analysis showed that the RNA segments of the swine isolates all came from avian isolates. A Bayesian phylogenetic analysis of a Chinese swine isolate revealed that it was a multiple reassortant, with its gene segments coming from avian H5N1, H9N2 and influenza viruses of other unknown subtype [92]. In Indonesia, multiple introduction events of the viruses from avian hosts to pigs have also been described [94].

### 3.3 Transmission of HPAI H5N1 to other Mammals

HPAI H5N1 has been also reported to infect other mammals apart from humans and pigs. These hosts include tiger [95-97], leopard [95], domestic cat [98-101], civet [102], dog [103-105], stone marten [106], pika [107] and donkey [108]. Phylogenetic analysis of the viral genome sequences has also been used to trace the origin of these viruses. For example, the H5N1 virus responsible for the cat infection was circulating among avian hosts in Thailand in early 2004 [98]. Phylogenetic analysis of the full genome sequence of the virus from civet revealed that it was closely related to HPAI H5N1 viruses of genotype G [102]. Similarly, the dog that died of HPAI H5N1 infection was also infected by viruses circulating in Thailand contemporaneously [104]. Therefore, most of these viruses in non-avian species were closely related to the AI isolates circulating in the region at the same time. However, a notable exception was that viruses from Raccoon Dogs from China were of genotype V, which was not the dominant genotype in China at that time [105]. 

## 4 INTER-SUBTYPE REASSORTMENT OF HPAI H5N1

Inter-subtype reassortment also plays an important role in the evolution and variation of HPAI H5N1 and has been frequently detected. It was reported that the internal genes of the HPAI H5N1 viruses in Hong Kong in 1997 were obtained from viruses of H9N2 subtype *via *inter-subtype reassortment [15]. Phylogenetic analysis of the eight separate segments has identified many H5N1 genotypes since 1996, such as A, B, C, D, E [7], V, W, X0-X3, Y, Z, Z^+^ [8], G [9]. Among them, the prototype HPAI H5N1 virus, Gs/GD/1/96 [1], reassorted with viruses of one or more unknown subtypes gave rise to genotypes A, B and C in 2000 [7, 8]. Viruses of genotype D obtained their NP gene from Dk/HK/Y280/97-like virus (H9N2 subtype) [7, 8]. The NP gene of genotype E virus came from viruses of an unknown subtype [7, 8]. Reassortment between genotype E and other aquatic AI viruses created genotypes X0-X3, whose PB2, PA and NS genes were not of H5N1 subtype [8]. Genotypes V, W, Y, Z and Z^+^ were also inter-subtype reassortants with some of the internal genes coming from aquatic AI viruses of other subtypes [8]. In addition, there are some genotypes that have been reported but not nominated. For example, viruses isolated from tree sparrows from Henan Province, China in 2004 belonged to a novel genotype [109]. Phylogenetic analysis revealed that they were created by inter-subtype reassortment between genotype A and AI viruses of other subtype.

In a few cases, inter-subtype reassortment and inter-host transmission occur together and create novel reassortants. In 2003, a HPAI H5N1 virus was isolated from pigs from Shandong Province, China. Phylogenetic analysis revealed that the M and NS gene came from H9N2 subtype and the PB1, PA and NP gene came from AI viruses of other subtypes [92].

## 5 TECHNICAL LIMITATIONS IN CURRENT ANALYTICAL METHODS

Phylogenetic analysis of viral sequences is a standard way to help delineate the molecular epidemiology of outbreak events, including those involving HPAI H5N1 viruses. However, the large numbers of sequences that are analyzed can sometimes cause particular problems. As of May 16^th^, 2011, there have been 602 virus genomes, 3,586 HA genes and 2,629 NA genes of H5N1 subtype available in GenBank [13]. With recent advances in sequencing technology, decreases in sequencing cost and the increased use of sequencing for epidemiological purposes, these datasets will become increasingly large. In this case, a traditional phylogenetic analysis becomes extremely difficult due to computational demands or problems with visualization of the outputs. Although there have been fast and paralleled algorithms to construct phylogenetic trees, such as PhyML [110] and RaxML [111], it is still hard to analyze a tree derived from a large dataset.

Proteotyping has been proposed to study the evolution of type A influenza [112, 113]. Proteotying is similar to genotyping at the DNA level and is able to capture the amino acid variations of the viruses [112,113]. It was used to analyze 2,196 AI virus genes and 169 complete virus genomes [112]. However, due to lack of computer programs to facilitate the proteotyping process and lack of appropriate standard to define proteotypes, this method has not been widely accepted by virologists thus far.

An alternative approach to phylogenetic analysis is to carry out “ordination” using a dimension reduction technique such as multidimensional scaling (MDS) [114], which is able to deal with very large numbers of sequences in a short period of time. MDS has been used to visualize antigenic variation in human influenza A, subtype H3N2 viruses [115]. Principal co-ordinates analysis (PCOORD) is equivalent to MDS when the distances are Euclidean. PCOORD has been used by us [85] to study the phylogenetic diversity of influenza A viruses and the accompanying software has also been used to analyze HBV and HCV sequence variation [116]. However, although PCOORD and other MDS techniques can yield information on the major groupings (similar to lineages for a phylogenetic tree) of the sequences and can analyze a large number of sequences, they fail to reveal ancestor-descendant relationship which can be easily seen from the phylogenetic tree.

Therefore, none of the methods currently available is suitable to analyze a large number of viral sequences. This has been proven to be problematical not only in the molecular epidemiology of influenza, but also in HIV, HBV, HCV and measles for example where there have been thousands of sequences available. To resolve this problem, novel mathematical techniques should be introduced and better computer programs should be developed.

In addition, several online or stand-alone computer programs have been developed to facilitate the genotyping and identification of reassortant influenza viruses [117-121]. Among them, FluGenome [117], the method proposed by Suzuki (2010) [119], GiRaF [120] and FluReF [121] are based on phylogenetic trees but employ different methods to determine the topological differences among the trees constructed using each genomic segment, while the quantitative genotyping algorithm developed by Wan *et al.* (2007) is not phylogeny-based [118]. Although these methods are robust and efficient, they are not extensively applied to date for the following reasons. Firstly, phylogeny-based programs are often time-consuming requiring computing power and therefore cannot deal with large datasets, although GiRaF is reported to be able to perform a large-scale analysis [120]. Secondly, although these methods generate consistent outputs, the results from each of them are not directly comparable and are therefore difficult to compare. For example, the authors of FluGenome analyzed ca. 2300 complete genomes of type A influenza and identified 156 unique genotypes [117]. However, Wan *et al.* (2007) identified 107 niches among only 283 complete genomes of H5N1 AI viruses [118]. In addition, GiRaF was also used to analyze 1101 whole-genome sequences of non-human H5N1 influenza viruses and identified 18 reassortment events [120]. Therefore, results obtained using these programs are not always consistent and this makes the explanation of the results more complicated. Lastly, the widely accepted nomenclature system for HPAI H5N1 is not employed by any of these computer programs.

## 6 FUTURE CHALLENGES FOR USING THE GENOMICS

Recent large-scale genome sequencing of HPAI H5N1 viruses, antigenic typing and database information mining have significantly improved the study of HPAI virus origin, diversity, transmission, reassortment and evolution. Future in depth studies of the influenza reservoir, along with large-scale data mining of genomic resources and the integration of epidemiological, genomic, and antigenic data, should enhance our understanding of antigenic drift and improve the detection and control of the emerging novel strains [12].

Genomic tools, such as high-throughput sequencing, viral and host mRNA and microRNA expression profiling, and microarray-based analysis of pathogen and host single nucleotide polymorphisms will prove to be important methods not only in revealing the patterns of circulation but also the mechanisms of the pathogenesis of HPAI H5N1 viruses among wildlife populations. Furthermore, these innovative new technologies may also help to identify the leads for therapeutic intervention, and to predict the new emergence of novel genotype/pathotypes with altered virulence and most importantly aid the development of effective vaccines [11].

To sum up, rapid identification of newly emerging viruses through the use of genomics tools is one of the major challenges in the near future. Phylogenomic and phylogeographic approaches combined with host-vector diversity, behavior and ecology will be more effective tools in tracing the origin, transmission, reassortment and evolution of the HPAI H5N1 viruses.
